# Monte Carlo Simulations for the Analysis of Non-linear Parameter Confidence Intervals in Optimal Experimental Design

**DOI:** 10.3389/fbioe.2019.00122

**Published:** 2019-05-24

**Authors:** Niels Krausch, Tilman Barz, Annina Sawatzki, Mathis Gruber, Sarah Kamel, Peter Neubauer, Mariano Nicolas Cruz Bournazou

**Affiliations:** ^1^Department of Bioprocess Engineering, Department of Biotechnology, Technische Universität Berlin, Berlin, Germany; ^2^Department of Energy, Austrian Institute of Technology GmbH, Vienna, Austria; ^3^DexLeChem GmbH, Berlin, Germany

**Keywords:** Monte Carlo, design of experiments, variance analysis, modeling, dynamic processes

## Abstract

Especially in biomanufacturing, methods to design optimal experiments are a valuable technique to fully exploit the potential of the emerging technical possibilities that are driving experimental miniaturization and parallelization. The general objective is to reduce the experimental effort while maximizing the information content of an experiment, speeding up knowledge gain in R&D. The approach of model-based design of experiments (known as MBDoE) utilizes the information of an underlying mathematical model describing the system of interest. A common method to predict the accuracy of the parameter estimates uses the Fisher information matrix to approximate the 90% confidence intervals of the estimates. However, for highly non-linear models, this method might lead to wrong conclusions. In such cases, Monte Carlo sampling gives a more accurate insight into the parameter's estimate probability distribution and should be exploited to assess the reliability of the approximations made through the Fisher information matrix. We first introduce the model-based optimal experimental design for parameter estimation including parameter identification and validation by means of a simple non-linear Michaelis-Menten kinetic and show why Monte Carlo simulations give a more accurate depiction of the parameter uncertainty. Secondly, we propose a very robust and simple method to find optimal experimental designs using Monte Carlo simulations. Although computational expensive, the method is easy to implement and parallelize. This article focuses on practical examples of bioprocess engineering but is generally applicable in other fields.

## Introduction

Only a few of the molecules developed in biotechnology are entering industrial production (Neubauer et al., 2013). In this regard, two big problems are being faced: (1) the long time to market due to the technical limitation of performing the high amount of necessary screenings, and (2) the high costs to perform those experiments. Hence, novel tools to shorten product development times are required in biomanufacturing. With this in mind, many biotech laboratories have been equipped with high throughput (HT) robotic facilities which perform a high number of very sophisticated experiments in parallel (Nickel et al., 2017; Hemmerich et al., 2018). Additionally, the implementation of computer aided tools for (semi-) automated experimental design is a complementing approach to exploit the full potential of modern technology, regarding not only hardware but also software (Glauche et al., 2017; Sawatzki et al., 2018). Nevertheless, especially in terms of a more consistent developmental path from small to industrial scale, dynamical experiments are required to investigate bioprocess performance, making dynamical models that describe the systems a non-negligible prerequisite.

The use of mathematical models to understand, describe, and predict natural phenomena is well-established in science (Tarantola, 2005). In engineering, large dynamical non-linear systems are designed and optimized, using advanced optimization algorithms and accurate mathematical models that describe the process (e.g., a large refinery, a complex electrical network, large metabolic networks). By this means, we can find the combination of inputs that for example maximizes profit while complying with social, environmental, and safety restrictions (Stephanopoulos and Reklaitis, 2011). Nevertheless, the solution obtained by the computer can only be as accurate as the mathematical model describing the real system (Velten, 2009). Experimental data is needed to ensure model accuracy. Models which are not sufficient in describing a certain process might be improved by e.g., using a more complex/simpler model structure. Experiments should be designed to generate informative data, enabling to assess the prediction power of the model with the highest possible certainty.

If the aim is to generate data to fit dynamical models, it is essential to apply model-based design of experiments (MBDoE; Körkel et al., 2004; Franceschini and Macchietto, 2008; Pronzato and Pázman, 2013). When dealing with non-linear processes, classical Design of Experiments (DoE) leads to a lower information content and thus to a higher variance of the parameters compared to MBDoE (van Daele et al., 2017). Originally, methods for Design of Experiments (DoE) have been developed in statistics (Box et al., 1978). Here, the goal is to design an experiment in such a way that an unknown system (black box) is understood as good as possible. An important assumption made in the regression models used for DoE is that the search space is small enough to allow a quadratic regression to describe the interaction between inputs (factors) and outputs (responses) of the system properly. A crucial characteristic of the regression models used in DoE is that they are linear with respect to their parameters, but this is not the case in more complex systems. A typical example of systems that are non-linearly dependent on the parameters are non-linear dynamical processes (e.g., most bioprocesses). In such cases, MBDoE (also known as Optimal Experimental Design) deals with the challenge of finding the experimental setup that minimizes the uncertainty on the parameter estimates of models that show a non-linear dependency on the parameters (Franceschini and Macchietto, 2008). This problem is not new and there has been an extensive work on it (Walter and Pronzato, 1990). The main goals of MBDoE are designing experiments such that: (i) the parameters of the model can be estimated with the highest accuracy, and/or (ii) the probability of selecting the best model structure is maximized (Kennard and Stone, 1969). This article focuses on the first one, as it is the more important and wider used application.

The use of MBDoE in combination with High Throughput facilities has been proven to drastically increase the efficiency of experimental campaigns (Cruz Bournazou et al., 2017; Barz et al., 2018). Yet, there remain some issues related to highly non-linear systems in combination with low information content of the experimental data. The main obstacle lies in the fact that the confidence intervals of the parameter estimates are approximated using the FIM, neglecting the effect of non-linearities in the model. Although this has been widely addressed in literature (Balsa-Canto et al., 2001; Raue et al., 2009; Kreutz et al., 2012), Monte Carlo Sampling is still the most accurate method for quantification of parameter uncertainty (Moles et al., 2003; Sin et al., 2009; López et al., 2015) after parameter estimation. In this work, we present the advantages and drawbacks of designing experimental inputs using linear approximations of the confidence region vs. more accurate Monte Carlo methods. We will also focus on the problems that arise when non-linear systems are approximated, and the confidence intervals are not well-represented by the ellipsoid confidence interval as predicted by the FIM. Finally, we propose a simple but robust method to improve the experimental design; especially useful in the initial steps when the parameter estimates are far from their true value and the experimental data is scarce. This may lead to an extremely large variance or covariance of the parameters, resulting in an ill-posed parameter estimation, mathematically represented by a close to singular FIM which is therefore not useful for MBDoE calculations. The main advantage of this alternative criterion for information content is, however, that it can be used with very limited understanding of MBDoE. We first present a model of a Michaelis-Menten reaction as an illustrative example, followed by a real case study of a biocatalytical reaction.

## Michaelis-Menten Kinetics as an Example in the Context of Model-Based Design of Experiments

### MBDoE for Parameter Estimation

In this section we present a short introduction to the basics of MBDoE. The reader is referred to Körkel et al. (2004); Franceschini and Macchietto (2008); Goujot et al. (2012) for a deeper insight into MBDoE. To this aim, we will follow a short introduction with an illustrative example that considers a reaction governed by the well-known Michaelis-Menten kinetics (Heineken et al., 1967).

### Problem Formulation

The generalized formulation of the problem is:

(1) $$\begin{array}{l} ẋ\left(t\right)=\frac{dx}{dt}=f\left(x\left(t\right),\;u\left(t\right),\theta \right)\;,\text{ with }x_{0}=x\left(t=0\right)\\ \hat{y}\left(t_{i}\right)=\;g\left(x\left(t_{i}\right)\right)+\eta_{i} \end{array}$$

where ẋ(*t*) denotes the vector of the time-derivates of the state variables in respect to the time *t* ∈ [*t*_0_, *t*_*end*_] ⊂ ℝ, $u\left(t\right)\in {\mathbb{R}}^{N_{u}}$ the time-varying inputs, $\theta \in {\mathbb{R}}^{N_{p}}$ the parameters of the model and η_*i*_ is assumed to be a random error, following a Gaussian independent and identical distribution (iid) with zero mean.

In general, MBDoE aims to find optimal experimental conditions, leading to the highest information content. The information content of an experimental run is affected by inputs (e.g., feed, pH), sampling (e.g., frequency, time points, number) and measurement error. We will focus on optimal sampling time computations throughout this work for the sake of clarity. Especially in dynamic processes an important factor to consider are the actual sample times, i.e., the time points when measurements are taken (Skanda and Lebiedz, 2010). Since the sensitivity of the outputs with respect to changes in the parameters changes over time, samples at specific time points contain a higher amount of information than others (Yue et al., 2006) and finding them is not always trivial. In complex biological experiments, experiments are expensive and time consuming and improving the data quality through optimal sampling points is essential.

### Numerical Optimization of the System of Ordinary Differential Equations

In an effort to demonstrate the performance with the most popular license free tools, the results presented were obtained applying scripts written in Python 2.7 (MBDoE-python). The gradients for the sensitivities (i.e., derivatives of model equations *f*(*x*(*t*), *u*(*t*), θ) w.r.t. *x* and θ) were calculated using sympy, the library for symbolic mathematics, the numerical solution of the system of ODEs (Vassiliadis et al., 1999) was performed using the sundials CVODE solver and the optimal experimental design was implemented using the optimize minimize solver (using the sequential least squares programming algorithm) of the package scipy.

### Parameter Estimation

The fitting function of the estimation problem is formulated based on the differences between the model predictions $y\left(\hat{\theta }\right)$ and the measured data ŷ for a specific set of parameters $\hat{\theta }$. Starting with an initial guess of the parameters, assessment of the new parameters is performed by calculating the squared sum of residuals and dividing it by the number of measurements as well as the individual variances σ^2^, i.e., the variance of the replicates at a specific measurement point (Bard, 1974), the so called maximum likelihood criterion:

(2) $$\begin{array}{l} J=\frac{1}{\sigma^{2}}*\frac{\sum_{k=1}^{N}{\left[y\left(\hat{\theta }\;\right)-\hat{y}\right]}^{2}\;}{n_{mes}} \end{array}$$

### Approximating the Confidence Interval Using the Fisher Information Matrix

**Figure 1:**
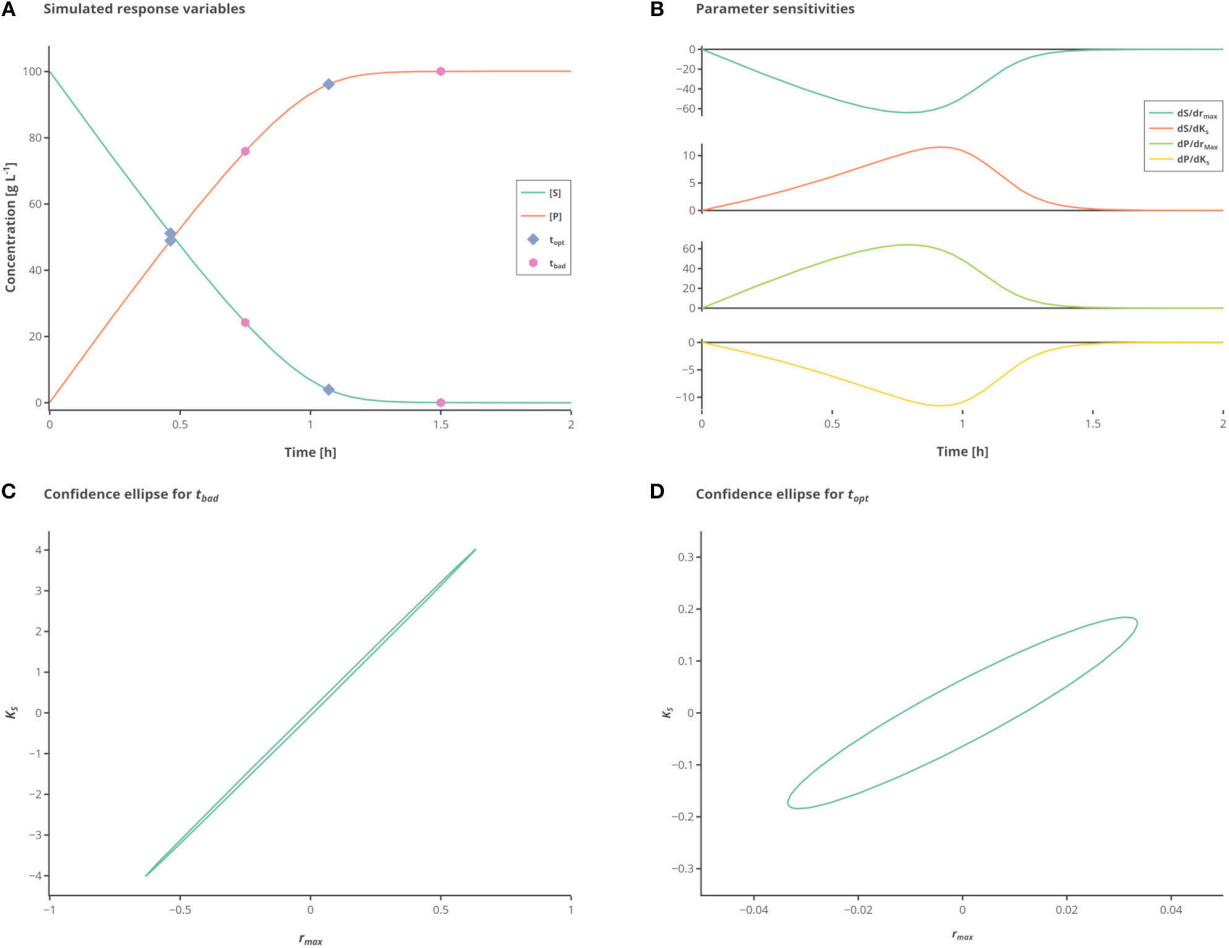
Influence of different measurement time points. **(A)** Simulated state variables. The state variables substrate (*S*) (green line) and product (*P*) (orange line) as well as the best (hexagon) and standard (circles) measurement points are shown over time. **(B)** Sensitivities of the state variables to the respective parameters. **(C)** Confidence ellipse for the unoptimized (“standard”) measurements. **(D)** Confidence ellipse for optimized (“best”) measurements. Interactive versions of these plots can be found at: https://www.tu-berlin.de/?204660.

The confidence intervals of the parameters can be visualized graphically for a certain confidence level (usually 90% or 95%). For a mathematical model with two parameters, the result is a confidence ellipsoid (Figures 1C,D). Commonly, first-order sensitivity analysis is applied to the problem and the resulting linearized confidence regions are examined to determine the accuracy of the parameter estimation problem. The computational burden is drastically reduced by approximating the confidence regions using the FIM. However, especially in the case of highly non-linear models, the linearized regions may not adequately represent the actually confidence intervals (Kostina and Nattermann, 2015). There are a number of methods that tackle this issue using methods like bootstrapping or sigma point. For a deeper insight, the reader is referred to Schenkendorf et al. (2009, 2018); Kreutz et al. (2012); van Daele et al. (2017). Nevertheless, these tools are quite complex and difficult to implement so that using the FIM remains the most used approach. The FIM is calculated using the sensitivities and the inverse of the covariance matrix of the measurement noise Σ (Guisasola et al., 2006):

(3) $$\begin{array}{l} FIM\left(\hat{\theta },\varphi \right)=\sum_{\text{k}=1}^{{\text{n}}_{exp}}\left[\frac{\text{d}\bar{\text{S}}}{\text{d}\text{t}}*\Sigma_{\hat{\theta },\text{k}}^{-1}\;*{\frac{\text{d}\bar{\text{S}}}{\text{d}\text{t}}}^{\text{T}}\right] \end{array}$$

The FIM is an indicator of the amount of information contained in the experimental data, as the inverse of the FIM is the Cramér-Rao lower bound of the unbiased estimation of the parameter variance-covariance matrix (Oliver Lindner and Hitzmann, 2006):

(4) $$\begin{array}{l} COV\geq \;FIM^{-1} \end{array}$$

Generally speaking, high values of the FIM lead to low approximate standard errors and thus low uncertainties of the estimated parameter values. It is therefore desirable to optimize some characteristic of the FIM in order to obtain most accurate estimations, which is done in the process of MBDoE.

In complex models with many parameters, difficulties in the parameter estimation and model validation arise, e.g., due to over-parameterization, insufficient quantity and quality of the experimental data as well as correlations between the model parameters. It may not be possible to determine all parameters with enough precision and accuracy (e.g., the variance exceeds some predefined threshold), which is known as parameter non-identifiability and an issue that has been widely addressed in literature (Holmberg, 1982; Vanrolleghem, 1995; Raue et al., 2009; Muñoz-Tamayo et al., 2018). Identifiability analysis can be performed to study the structural and local properties of the model (Audoly et al., 2001; Kreutz et al., 2012; Kravaris et al., 2013; López et al., 2013) and can further be improved if the experiments are properly designed. Some parameters can only be estimated if there are enough measurements or if the experimental design is not correlated.

### Model-Based Experimental Design for Parameter Estimation

Model-based experimental design for parameter estimation aims at reducing the confidence regions by modifying the experiment design vector φ. The experiment design vector holds all the values describing the prospective experiment, i.e., the external stimuli *u*(*t*), such as the experimental duration, sampling times *t*_*i*_ and initial values *x*_0_. Since a measurement set is selected here, the only variables in the design vector are the different sampling times *t*_*i*_.

To yield a new experiment design vector φ^*^ for an optimally designed experiment, the FIM Ω must be maximized (Yu et al., 2015):

(5) $$\begin{array}{l} \varphi^{*}=\arg\underset{\varphi \in \Phi }{\max}\Omega \left(FIM\left(\theta ,\varphi \right)\right) \end{array}$$

To perform optimization, i.e., maximization of the FIM, the FIM needs to be transferred to a scalar target criterion. The most common target criteria in the context of MBDoE are the D-optimal, E-optimal and A-optimal criteria (Silvey, 1980; Franceschini and Macchietto, 2008). Table 1 briefly summarizes their characteristics.

**Table 1:** Characteristics of the standard optimal experimental design criteria.

**Criterion**	**Aim regarding the FIM**	**Aim regarding the joint confidence region**	**Advantage**	**Disadvantage**	**Calculation**
D-optimality	Maximizing the determinant	Minimizing the volume	Easy graphic interpretation	Gives great importance to parameter to which the model is most sensitive	det(FIM)
E-optimality	Maximizing the smallest eigenvalue	Minimizing the size of its major axis	Positive effect on parameter correlation	No continuous function	eigenvalue_min_(FIM)
A-optimality	Minimize the trace of the inverse	Minimizing the dimensions of the enclosing box	Low computational effort	Less accurate in case of high cross-correlation between parameters	tr(FIM^−1^)

Each of the different design criteria has its advantages and disadvantages. The commonly used D-criterion can be easily graphically interpreted as it equals the area of the joint confidence region and is theoretically invariant to rescaling. However, the D-criterion comes with the disadvantage of giving great importance to the most sensitive parameter and may show an increase in the parameter correlation, even though the total confidence region shrinks (Zullo, 1991). By minimizing the size of the major axis of the joint confidence region, optimization using the E-criterion shows a positive effect on the parameter correlation, but it might be impossible to find an optimal solution, since the E-criterion is not a continuous function (i.e., it might be impossible to use gradient-based search methods; Körkel et al., 2004). The A-criterion has the advantage of being easily calculated by only adding up the parameter variances. However, in case of high cross-correlation between the parameters, this criterion is disadvantageous, due to neglection of the off-diagonal elements of the variance-covariance matrix.

Further possibilities regarding the optimization process are robust criteria for design of experiments which are less sensitive to the initial values used for parameter estimation, e.g., the maximin design for optimizing the worst possible performance of any value θ in the parameter space (Körkel et al., 2004; Chen et al., 2017; van Daele et al., 2017; Telen et al., 2018).

Some of the most recent examples for model-based design of experiments in biotechnology are summarized in the Supplementary Material, introducing standard and special criteria as well as showing their application in daily research.

### Designing an Optimal Experiment for a Michaelis-Menten Reaction

The benefits of performing MBDoE are demonstrated using a well-known reaction in biochemistry, the Michaelis-Menten kinetics. This kinetics (Heineken et al., 1967) describes the enzyme-catalyzed conversion of a substrate (*S*) to a product (*P*) via an enzyme substrate complex (*ES*). The reaction rates *k*_1_ and *k*_−1_ represent the rate of enzyme substrate complex formation and dissociation, whereas *k*_2_ is the rate of product formation.

(6) $$\begin{array}{l} E+S\begin{array}{c} k_{1}\\ ⇌\\ k_{-1} \end{array}ES\overset{k_{2}}{\to }P+E \end{array}$$

The system of ordinary differential equations (ODEs) describes the dynamic changes in the substrate $\frac{dS}{dt}$ and product concentration $\frac{dP}{dt}$ as a function of the unknown parameters *r*_*max*_ (maximum specific reaction rate) and *K*_*s*_ (half saturation constant) as well as *S*, the substrate concentration:

(7) $$\begin{array}{l} \frac{dS}{dt}=-r_{\max}*\frac{S}{S+K_{S}},with\;K_{S}=\frac{k_{-1}+k_{2}}{k_{1}}\\ \frac{dP}{dt}=-\frac{dS}{dt} \end{array}$$

The measured values *S* and *P* are subject to iid zero-mean Gaussian-distributed measurement errors. In this case, there are two parameters to fit which are strongly correlated unless observations are made showing the change on the reaction rate with respect to substrate consumption. By simply choosing proper time points for the samples the information gained from a single experiment with two measurements can be drastically increased. To better illustrate the large differences in the confidence intervals which are due to the different sampling points, the experimental data is only measured 2 times, as depicted in Figure 1A.

While the sensitivities of the respective parameters are depicted in Figure 1B. Figure 1C shows the confidence intervals in the standard scenario, relying on the data which is obtained by measuring at the measurement points *t*_*bad*_ (see Table 2), which represent good initial guesses when measuring two times over a period of 2 h. As can be seen by evaluating the confidence intervals, the maximal reaction rate *r*_*max*_ is determined with a higher precision than the half saturation constant *K*_*S*_. Although, because of the large confidence region it is nearly impossible to conclude the true parameter values. In Figure 1D the confidence region is shown after the optimization of the measurement set according to the A-optimal criterion (*t*_*opt*_). The variation, especially for *K*_*S*_ but also for *r*_*max*_, is reduced drastically, enabling a good estimation of the true parameter values. This highlights the importance of optimal sampling points to obtain data for parameter estimation.

**Table 2:** Concentrations for measurement times *t*_*opt*_ and *t*_*bad*.._.

***t* [h]**	***S* [g L^**−1**^]**	***P* [g L^**−1**^]**
***t**_***opt***_*		
0.463	51.145	48.955
1.070	3.940	96.160
***t**_***bad***_*		
0.750	24.192	75.908
1.500	0.033	100.067

## Monte Carlo Based Parameter Confidence

As mentioned before, the FIM is used to approximate the covariance of the parameters that are determined from fitting the experimental data to a model. This can be used in general to determine the degree of confidence for the estimated parameters. To calculate an optimality criterion (which can be used to identify optimal experimental settings), the FIM is commonly used in MBDoE. However, the FIM neglects the non-linearities of the model and it is only valid in the proximity of the optimal parameters.

Performing Monte Carlo simulations is a valuable alternative to compute the real non-linear confidence intervals of the parameters (Buckland, 1984; Alper and Gelb, 1990; Sin et al., 2009). In contrast to the FIM, which is directly linked to the covariance of the parameters, Monte Carlo simulations are based on repeatedly carrying out parameter estimations for the experimental data, which is perturbed by a random error. The error in this example is derived from a Gaussian distribution with μ = 0 and σ^2^ = 0.16, based on expert knowledge. The Monte Carlo simulations then yield new values for the respective parameters which can be plotted pairwise to obtain a graphical view of the parameter distribution. Considering the noise in the data and given a sufficient large number of simulations (in our case 5,000 runs deliver very accurate results, although 500 are usually enough in the context of MBDoE), the Monte Carlo will result in a better representation (i.e., actually showing non-linearities) of the real distribution of the parameter combinations, particularly for highly non-linear models. This will be shown in a later section.

In the following section it will be demonstrated that for the Michaelis-Menten example the FIM provides a very good approximation. In a second example—a more realistic challenge—it will be demonstrated how this approach fails and why alternatives, such as the Monte Carlo approach are required in order to validate the results.

### Accuracy of the Approximated Solution

**Figure 2:**
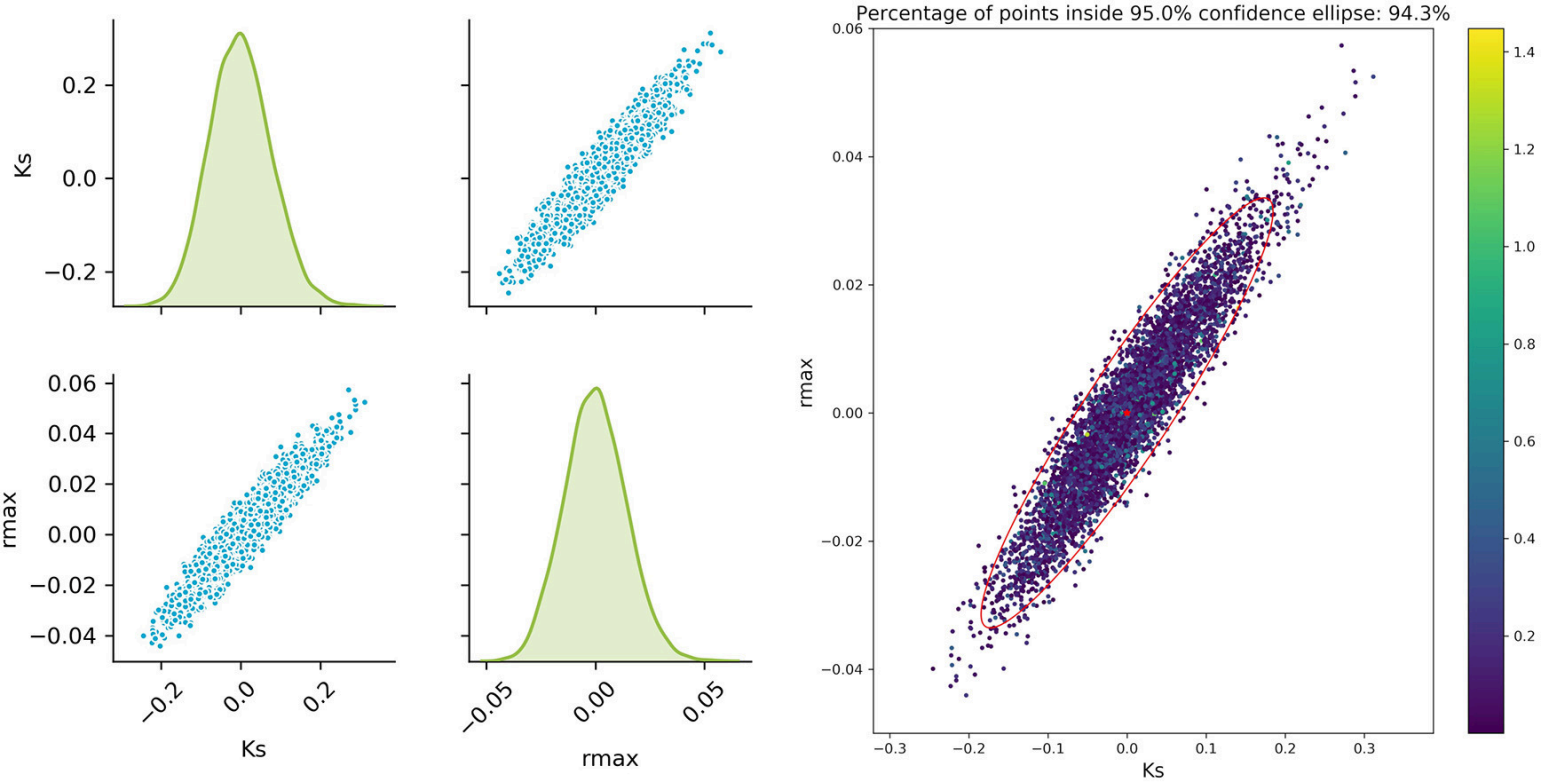
Monte Carlo results for *t*_*opt*_; **Left**: pairplots of parameters obtained with a data noise using μ = 0 and σ^2^ = 0.16; **Right** MC vs. FIM.

**Figure 3:**
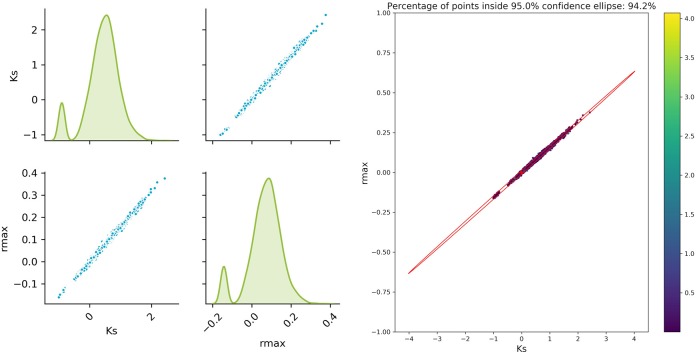
Monte Carlo results for *t*_*bad*_; **Left**: pair plots of parameters obtained with a data noise using μ = 0 and σ^2^ = 0.16; **Right** MC vs. FIM.

The sampled parameter distribution is shown in Figure 2. On the left-hand side, the distributions for the two parameters obtained with the Monte Carlo-Simulations (performing parameter estimation of data with added noise) as well as the corresponding scatterplots are shown. The scatterplot can be directly compared with the confidence ellipsis obtained from the FIM as illustrated on the right-hand side of Figure 2. It can be clearly seen that there is a very good agreement between the FIM and the Monte Carlo results for *t*_*opt*_. The Monte Carlo points also create an ellipsoidal shape and 94.3% of the points are inside the corresponding 95% confidence ellipse. Considering the measurement times *t*_*bad*_, the figure shows a reasonable agreement, however non-linearities already become visible (pairwise plot and comparison in Figure 3).

The experimental design is indeed improved by MBDoE, increasing the information content and thereby reducing the uncertainty of the parameter estimates. For this simple model, linearization to estimate the parameter confidence intervals delivers satisfying results even for the worst measurement points, as can be seen from the good agreement of the FIM and Monte Carlo sampling. Nevertheless, the results highly depend on the accuracy of our approximation of the parameter uncertainty and will fail to deliver correct prediction in problems that show a higher non-linearity. This can be seen in a real example of a biocatalytic reaction. In this example taken from a real case, the limited observations and understanding of the underlying phenomena make it extremely difficult to correctly predict the distribution of the parameter estimates and hence find the best design to minimize it.

## Case Study: Enzymatic Synthesis of Pentose-1P Using Thermostable Nucleoside Phosphorylases

α-D-pentofuranose-1-phosphates (Pentose-1Ps) are of increasing interest because of their metabolic, industrial and potential clinical significance (Tozzi et al., 2006; Kamel et al., 2018). With the world moving toward green chemistry, the development of efficient enzymatic synthesis process moved into the focus of scientists and researchers. Recently, the enzymatic synthesis of Pentose-1P using thermostable nucleoside phosphorylases was reported as a practical alternative to the chemical synthesis (Kamel et al., 2018).

Nucleoside phosphorylases are well-studied enzymes (Pugmire and Ealick, 2002; Yehia et al., 2017). They catalyze the revisable cleavage of nucleosides, in the presence of inorganic phosphate (P_i_), producing a nucleobase and Pentose-1P. The reaction speed and equilibrium are dependent on different factors including: (i) nucleoside concentration, (ii) phosphate concentration, (iii) the ratio between the nucleoside and the phosphate, (iv) enzyme concentration, (v) reaction temperature, and (vi) stability of the reactants and the products.

### The Biocatalytical Reaction of Interest

**Figure 4:**
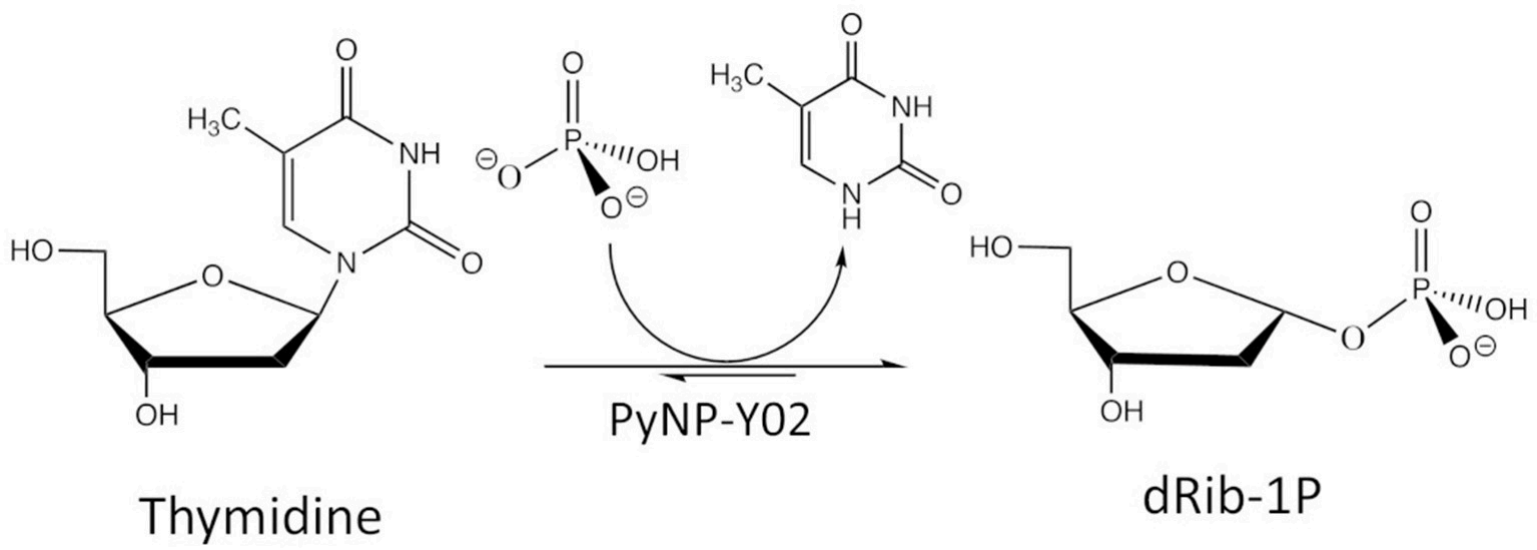
Reaction scheme of the biocatalytic conversion.

The case study considers 2-deoxy-α-D-ribofuranose-1-phosphate (dRib-1P) synthesis from thymidine (Thd) using thermostable pyrimidine nucleoside phosphorylase (PyNP). Thd is phosphorolytically cleaved producing thymine (Thy) and dRib-1P using thermostable PyNP-Y02 as shown in Figure 4.

To estimate the different parameters and examine the effect of the different factors on the enzymatic synthesis of dRib-1P, reactions were conducted at different conditions with varying concentrations of enzyme and substrate or changing the temperature of the reaction. Moreover, different ratios of substrate to P_i_ were tested, since reaction speed is thought to be affected by this (Kamel et al., 2018). Since many factors are affecting the enzymatic reaction and there are lots of possible levels for each factor, using the traditional approach to identify optimal conversion conditions would require hundreds of experiments, as well as lot of time and effort. Applying dynamic mathematical model offers a great chance for finding optimal conditions with a minimum number of experiments.

### Kinetic Model

It is assumed that the product is formed through a single enzyme-substrate-complex (*Enz*_*comp*_). The reaction rates for complex formation are *k*_1_ and *k*_−2_ and for converting the complex into educts or products are *k*_−1_ and *k*_2_, respectively.

$$\text{Enz}+\text{Thd}+{\text{P}}_{\text{i}}\;\overset{k_{1}}{\underset{k_{-1}}{⇌}}{\text{Enz}}_{\text{comp}}\;\overset{k_{2}}{\underset{k_{-2}}{⇌}}\text{Thy}+\text{R1P}+\text{Enz}$$

The applied quasi-steady-state-approximation yields:

(8) $$\begin{array}{l} \text{Enz}={\text{Enz}}_{\text{t}=0}-{\text{Enz}}_{\text{comp}} \end{array}$$

(9) $$\begin{array}{l} \frac{d\left({\text{Enz}}_{\text{comp}}\right)}{dt}=0 \end{array}$$

With this approximation, the ODE system is simplified and the following equation for the product formation as well as substrate consumption rate are used:

(10) $$\begin{array}{l} v\;=\frac{{\text{Enz}}_{\text{t}=0}\left(k_{1}k_{2}\text{Thd}\cdot {\text{P}}_{\text{i}}-k_{-1}k_{-2}\text{Thy}\cdot \text{R}1\text{P}\right)}{k_{1}\text{Thd}\cdot {\text{P}}_{\text{i}}-k_{-2}\text{Thy}\cdot \text{R}1\text{P}+k_{-1}+k_{2}} \end{array}$$

This formation rate can also be written in the classical form of the reversible Michalis-Menten equation:

(11) $$\begin{array}{l} v\;=\;\frac{r_{\max}^{f}/K_{M,S\;}\;{-\;r}_{\max}^{r}/K_{M,P}}{1+\frac{\text{Thd}\cdot {\text{P}}_{\text{i}}}{K_{M,S}}-\frac{\text{Thy}\cdot \text{R}1\text{P}}{K_{M,P}}} \end{array}$$

The maximum rates *r*_*max*_ and Michaelis-Menten constants *K*_*M, i*_ are used which can be derived from the reaction rates *k*_*i*_ from above.

(12) $$\begin{array}{l} K_{M,S}\;=\frac{k_{-1}+k_{2}}{k_{1}},K_{M,P}\;=\frac{k_{-1}+k_{2}}{k_{2}^{{}^{\prime }}},r_{\max}^{f}\;=\;{Enz}_{t=0\;}\\ k_{catf}\;=\;{{Enz}_{t=0}\;k}_{2},\text{and}r_{\max}^{r}\;={Enz}_{t=0\;}k_{catr}\;=\;{Enz}_{t=0}\;k_{-1}, \end{array}$$

Furthermore, the temperature dependence of the reaction rates *k*_*i*_ is considered by the following approximation, which is similar to a Gaussian distribution, where *T*_*max*_ represents the optimal temperature and *T*_*width*_ the deviation (Szeker et al., 2012):

(13) $$\begin{array}{l} k_{i,T}\;=\;k_{i}e^{-\frac{{\left(T-\;T_{\max}\right)}^{2}}{{\left({2*T}_{width}\right)}^{2}}} \end{array}$$

Thus, for the final model the six model parameters *K*_*mS*_, *K*_*mP*_, *k*_*catf*_, *k*_*catr*_, *T*_*max*_, and *T*_*width*_ need to be estimated.

### Materials and Methods of the Case Study Example

To identify the reaction parameters of the mathematical model, different experimental conditions were tested as summarized in Table 3. Samples were taken at different time points. Experimental conditions were determined based on our preliminary data and suggestions from factorial DoE. The ranges of the respective concentrations were based on expert knowledge. All experiments were repeated twice, and 2 samples were taken and measured at each time point. Thymidine (Carbosynth, UK) was phosphorolytically cleaved by pyrimidine nucleoside phosphorylase (PyNP-Y02) (BioNukleo, Germany) in phosphate buffer. Thymidine and the formed Thymine were separated via HPLC using a reversed phase C18 column (150 × 4.6 mm) (Phenomenex, USA) as described previously (Szeker et al., 2012) and were quantified in reference to standards. The conversion to percent was calculated as following:

(14) $$\begin{array}{l} \text{Conversion }\left[\%\right]=\frac{{\text{c}}_{\text{Product}}\left(\text{Thy}\right)\left[\text{mM}\right]}{{\text{c}}_{\text{Product}}\left(\text{Thy}\right)\left[\text{mM}\right]+\;{\text{c}}_{\text{Substrate}}\left(\text{Thd}\right)\left[\text{mM}\right]}\times 100\% \end{array}$$

**Table 3:** Experimental design.

**Experiment**	**Thd. conc. [mM]**	**P_**i**_ conc [mM]**	**Enzyme conc. [mg/ml]**	**Temp [^**°**^C]**
1	100	250	0.005	40
2	100	250	0.117	40
3	100	250	0.117	50
4	100	250	0.117	60
5	100	750	0.117	50
6	50	500	0.230	60
7	50	750	0.050	40

### Identifiability Problem and Model Revision

**Figure 5:**
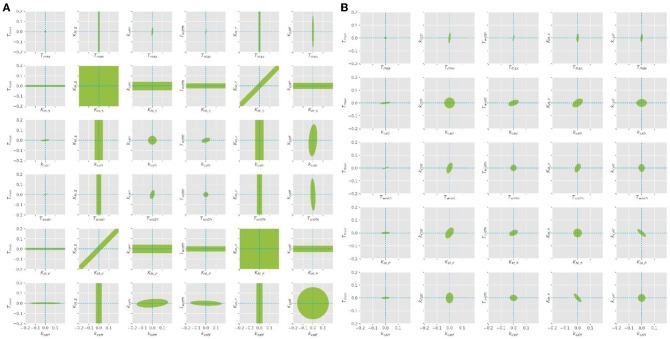
Confidence ellipses for the optimal parameters obtained with the quasi steady state assumption. **(A)** Leaving *K*_*M, S*_ free, **(B)** *K*_*M, S*_ is fixed at constant value of 1.3 as derived from literature.

The parameter estimation was performed using the experimental data obtained from the settings described in Table 3. A Latin hypercube sampling (LHS) design (Sacks et al., 1989) was used to generate initial parameter guesses which represent a reasonable coverage of the parameter search space. A gradient based search algorithm was used for each realization. The selected estimates correspond to the best solution obtained. While the parameters *T*_*max*_, *T*_*width*_, and *k*_*cat*_ seemed to be well-defined, it was found that different combinations of *K*_*M, S*_ and *K*_*M, P*_ parameters lead to comparable RSS values, since both parameters are highly correlated (cf. Figure 5A). A common way to solve this problem is to regularize the PE problem using some type of *a priori* information (Golub et al., 1999). Alternatively, the problem can be regularized based on expert knowledge to fix one of the parameters at a constant value and estimate the remaining parameters. Since literature values can only be found for *K*_*M, S,*_ this value was chosen to be fixed at 1.3, as suggested by the work of Szeker et al. (2012). This reduces the number of parameters which need to be estimated from the available data and drastically decreases the confidence intervals of the parameters, as can be seen in Figure 5B. It can be easily seen that when *K*_*M, S*_ is left free, the (joint) confidence regions are very large, thus it is not possible to estimate unique values for both parameters at the same time. However, when the value is kept constant, the confidence intervals shrink considerably.

### Experimental Results and Simulation

**Figure 6:**
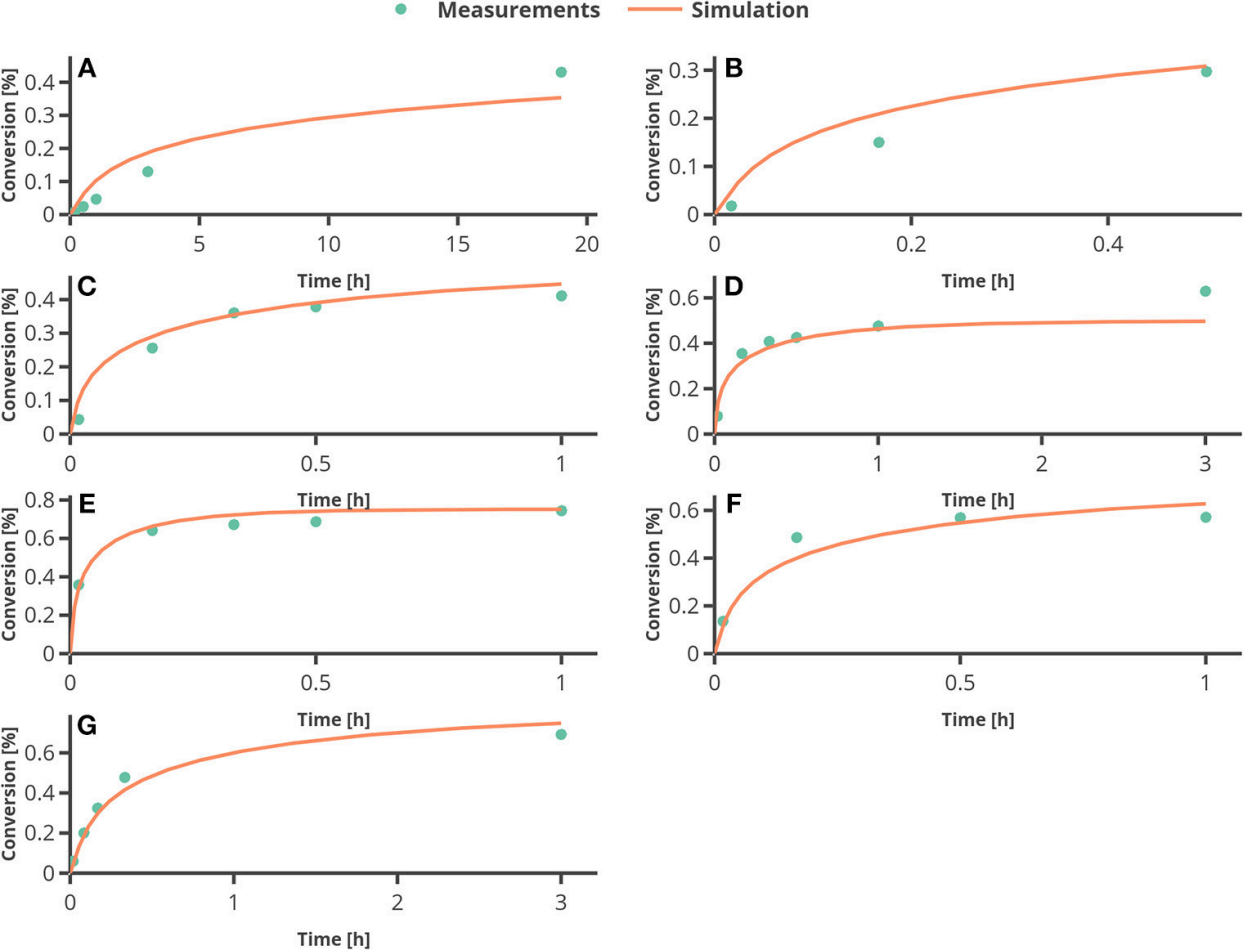
Results from fitting the model to experimental data. **(A–G)** represent the experimental conditions 1–7 as in Table 3.

In Figure 6, the percentage of conversion from substrate to product is plotted over time. The only larger deviation can be seen at the end of the reaction for the experimental setting 4 (see Figure 6D), where the temperature was set to 60°C. To assess the quality of the parameter estimation outcome, the confidence regions (obtained from the FIM) will be compared to Monte Carlo simulations.

### Accuracy of the Parameter Uncertainty

Using the FIM as an indicator for the accuracy of the parameters might lead to the false conclusion that the parameters are accurately estimated and can be used for further simulation as some scalar measure of the FIM reaches its optimum. However, due to the model's inherent non-linearities, the FIM is only a vague approximation of the actual uncertainties (since the FIM is based on a linear approximation) and reflects the parameter's uncertainties only in the vicinity of their optimal values. To obtain a more realistic insight into the parameter ranges, Monte Carlo simulations can be used for a more accurate analysis of the real parameter regions. These simulations offer a simple to implement, yet accurate method for the representation of the parameter's confidence regions. Researchers are therefore encouraged to perform Monte Carlo simulations, especially in cases where the model parameters are far from their optimal values. Even though the FIM and their scalar measures offer a quick validation of the parameter estimation outcome and can be used for simple cases, performing OED based on Monte Carlo simulations is recommended in bioprocess models, which usually show a higher degree of non-linearity. Especially when it comes to scenarios, where the FIM is non-invertible and therefore the classical criterions are not accessible, performing Monte Carlo simulations offer a simple method to perform further OED steps.

**Figure 7:**
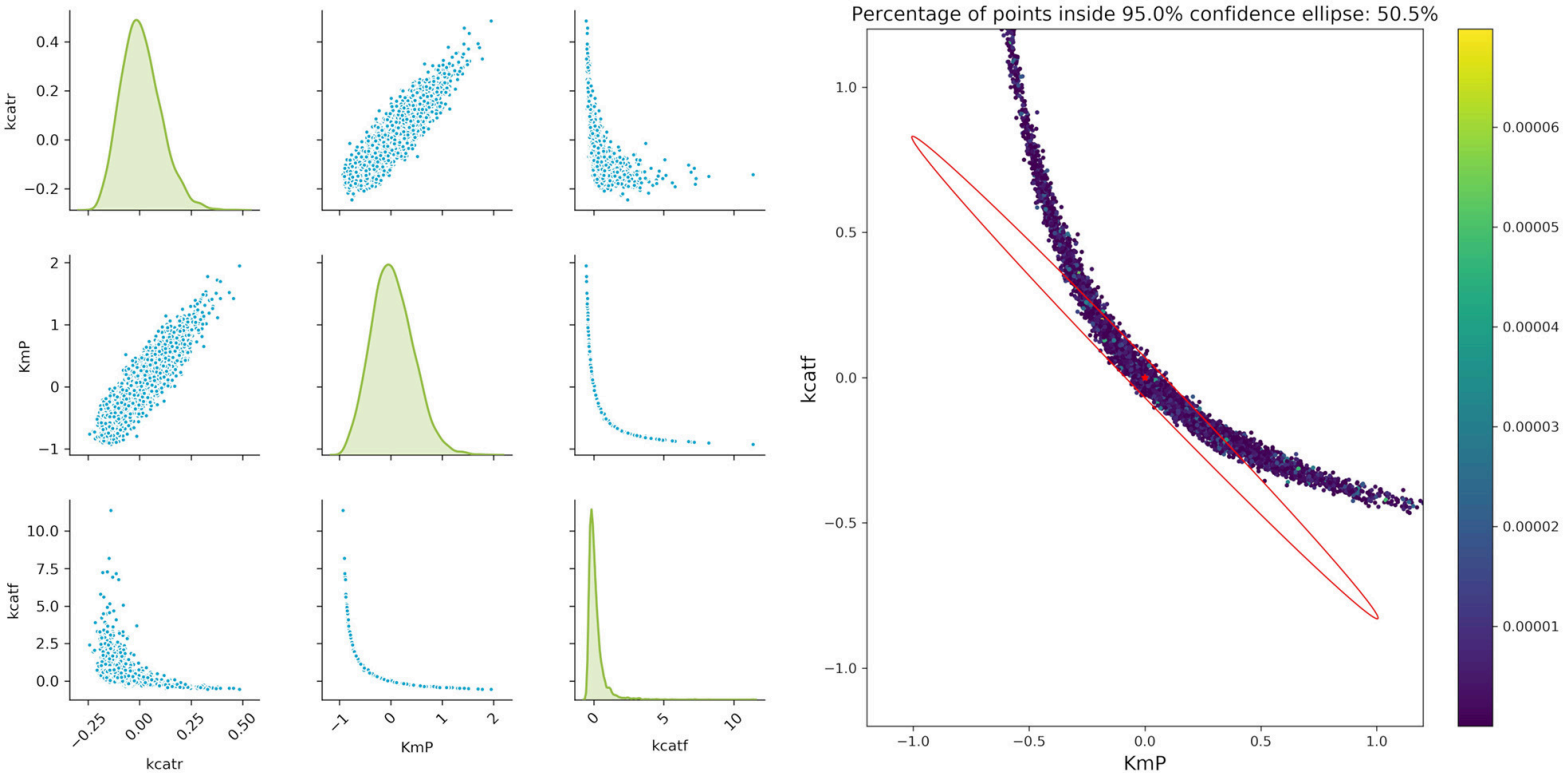
Monte Carlo results for biocatalytic example; **Left**: pair plots of parameters obtained with data perturbed with Gaussian distributed noise using μ = 0 and σ^2^ = 9*10^−6^; **Right** MC vs. FIM for one example, indicating the non-linearities of the parameter estimation.

Considering that we want to compute the following design with only two experiments at hand, the number of experiments will be reduced. Pairwise scatter plots of the parameters as well as comparison with the FIM show that the actual confidence intervals of the parameters strongly deviate from the ones estimated by the FIM as can be seen in Figure 7. Thus, regarding further model validation, it is essential to thoroughly verify the parameter estimation outcome. Especially nowadays, with easy and cheap access to large computational power (e.g., clusters or cloud computing), Monte Carlo simulations for estimating the parameter confidence intervals are a powerful alternative to traditional methods. This opens the possibility for researchers to easily assess their parameter estimation outcome, even for highly non-linear models, without utilizing mathematical tools which require a deeper understanding of the validation techniques.

An important factor when analyzing the confidence regions of the parameters is the non-linearity of the parameter confidence regions, which cannot be easily represented by the FIM ellipsoids. This is true even for experiments with a very high measurement accuracy (setting the artificial noise variance of the parameters to a value of 9 × 10^−6^). A closer analysis of the three more interesting parameters *kcatf* , *kcatr*, and *K*_*M, P*_ fitted to two theoretical experimental conditions (which are derived from conditions 5 and 7), where the time points were modified as in the aforementioned example shows that there is a strong non-linearity. While a pairwise analysis of *kcatf* and *kcatr* can still be approximated by the FIM, plotting the combination of *kcatf* and *K*_*M, P*_ is rather curve-shaped as depicted in Figure 7. Moreover, only around 50.5% of the Monte Carlo samples lie within the 95% confidence region predicted by the FIM. This indicates that Monte Carlo calculations give a better insight into the model's underlying non-linearity and actual parameter distribution.

### A New Scalar Design Criterion for Assessment of General Nonlinear Confidence Regions

Since most design criteria used for assessing the amount of information gained from certain experimental data rely on some scalar metric of the FIM, it is questionable to which extent they are applicable for non-linear models. Especially when fitting data to models showing high non-linearity, the parameter uncertainties might become too large and the FIM thus non-invertible. To tackle this issue and to quickly assess the outcome of Monte Carlo calculations, we propose a new criterion to quantify the variation in the data: The Q-criterion, the quantile related criteria. This criterion is comparable to the A-criterion, which adds up the variances of the parameters. Using quantiles as a measure for the variance in the data offers the advantages of an easy calculation, which is at the same time robust against outliers in the data. For the Q-criterion, the squared distance for every parameter *i* between the quantile_0.9_ (90th percentile of the Monte Carlo based parameter values) and quantile_0.1_ (10th percentile of the Monte Carlo based parameter values) is calculated as scalar measure for the variation in the data:

(15) $$\begin{array}{l} {\text{Q}}_{\text{crit}}=\underset{\theta_{\text{i}}}{\sum }{\left({\text{Q}}_{\theta_{\text{i}}\text{,0.9}}-{\text{Q}}_{\theta_{i},0.1}\right)}^{2} \end{array}$$

**Figure 8:**
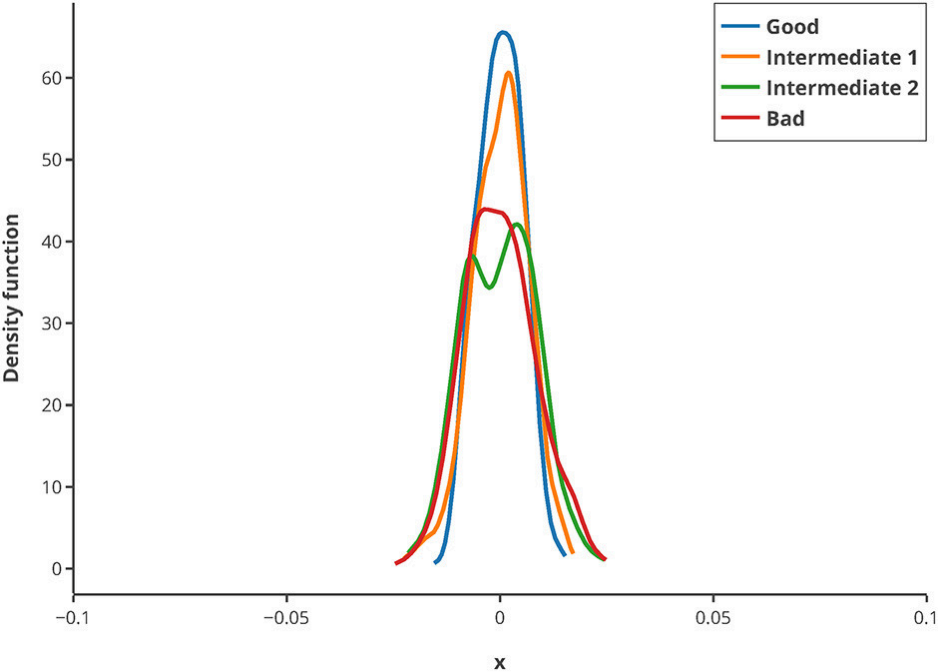
Kernel density estimation distribution of normalized Q-criterions for different samples and measurement qualities. The Q-criterion was calculated 500 times for 80% of the original sample pool and the obtained distribution normalized by its mean to show the robustness of the criterion. The legend shows the quality of sampling points.

To illustrate the usage of this criterion, *in silico* MBDoE was performed with iteratively improved measurement points, based on the best values of the Q-criterion in every round. The new criterion can be used to iteratively improve the measuring time points of an experiment as well as for model calibration. To assess whether the criterion is sufficiently robust regarding low sample size, 500 repeats of randomly drawn samples (correspond to 80% of the original sample pool) were used for calculating the Q-criterion and normalized by their mean. They showed a very low deviation from the mean (see Figure 8), and hence suggest that the Q-criterion is robust against small sample sizes. Hence, even for small data sets or outliers in the data, the criterion delivers good results.

Monte Carlo simulations give researchers who are not very experienced in the field of uncertainty analysis an easy to use tool for to assessing the outcome of parameter estimations. Especially when it comes to highly non-linear models in which second order derivatives cannot or not easily be calculated, Monte Carlo simulations and the Q criterion offer a great alternative to quickly improve the optimization outcome regarding MBDoE.

### Proof of Concept

**Figure 9:**
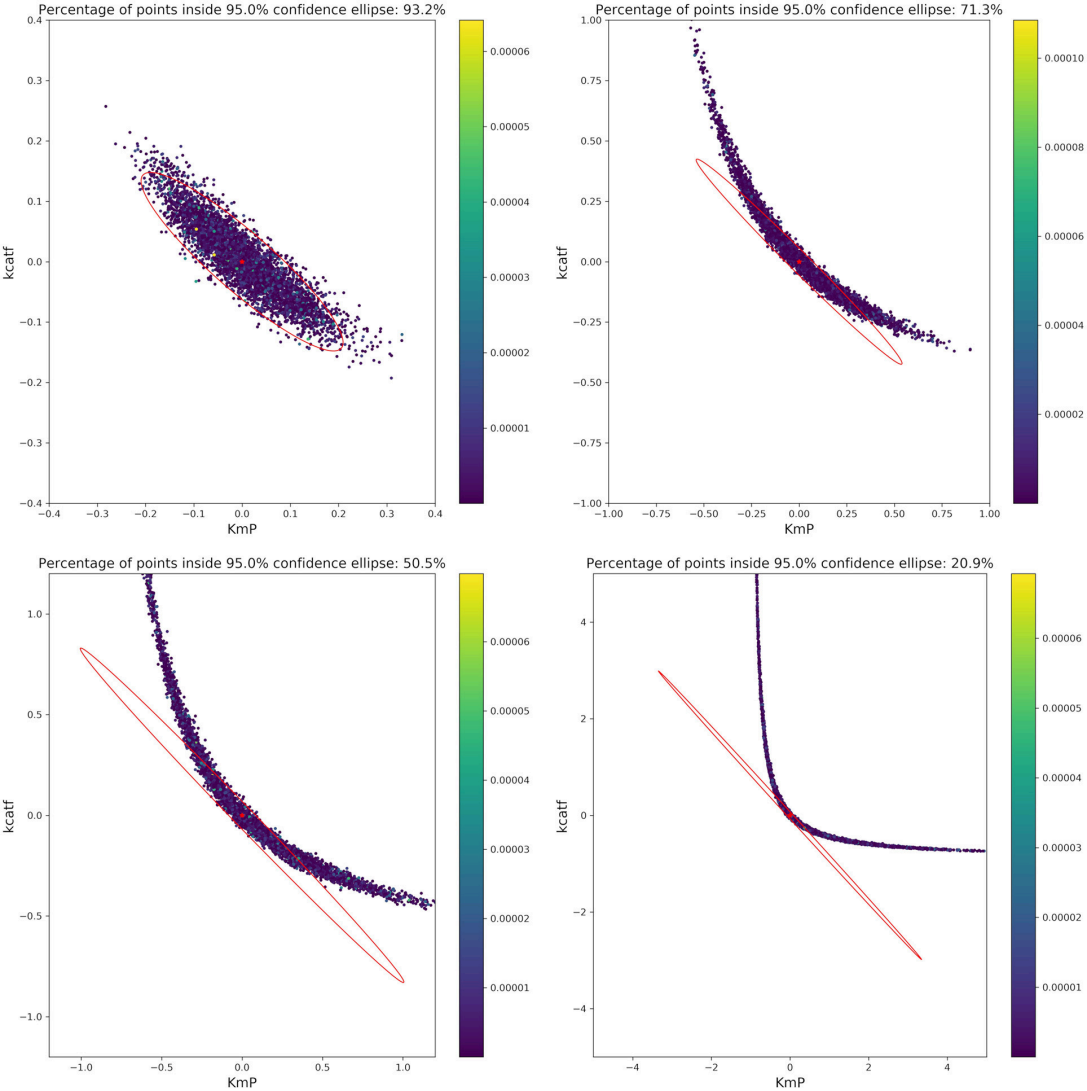
Visualization of the FIM and results from Monte Carlo simulations for two parameters for 4 different sample times (Good, intermediate 1, intermediate 2, bad, from top right to bottom left). The color bar indicates the individual cost (= LSQ) from that specific experiment. Especially for highly non-linear models, the FIM is a bad representation of the actual parameter values.

Based on the optimal parameters, *in silico* experimental data was generated at different time points for the biocatalytic example to reflect different qualities in the measurements. Subsequently, Monte Carlo simulations were performed at these time points. This was done in order to compare the confidence intervals obtained from the FIM with the results from the Monte Carlo simulations. By comparing the intervals (Figure 9) it can be concluded that the FIM is not sufficiently considering the model's non-linearity. The percentage of points from the Monte Carlo simulation overlap with the region obtained from the FIM only for the good sample points. For the other sampling points the FIM becomes only larger in size, while the Monte Carlo simulations also show a more non-linear distribution.

The infeasibility of the A-criterion to consider the model's non-linearity can be further underlined when looking at the Q-criterion at different sampling points. For the good and intermediate sampling points, the Q-criterion and the Q-criterion calculated for 5,000 samples derived from a multivariate Gaussian distribution with μ = 0 and Cov = FIM^−1^ deliver similar values, as can be seen in Table 4. However, it should be noted that the value of the Q-criterion for the bad sampling points is much worse when derived from the FIM as opposed to when derived from the Monte Carlo samples, showing that the FIM overestimates the variance of the parameters.

**Table 4:** Comparison of different optimization criteria for different sampling points.

**Sampling points**	**A-criterion**	**Q-criterion**	**Q-criterion with FIM based scatter**
Good	0.01	0.08	0.07
Intermediate 1	0.08	0.55	0.54
Intermediate 2	0.28	2.09	1.81
Bad	3.35	12.86	22.10

*Shown are the A- as well as the Q-criterion compared to the Q-criterion calculated for a multivariate Gaussian distribution with its covariance matrix derived from the FIM*.

**Figure 10:**
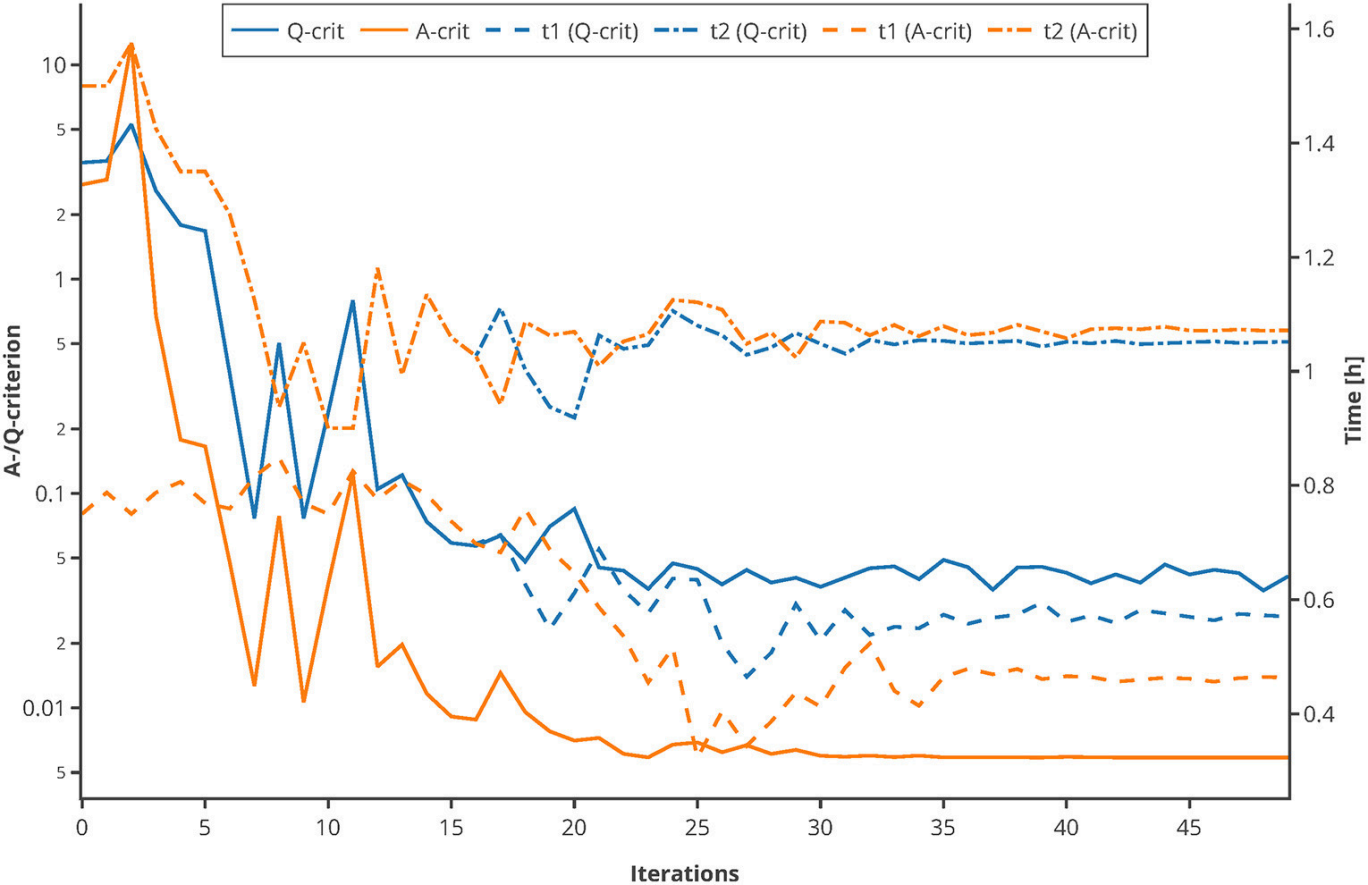
Comparison of A-/Q-criterion guided optimization of sampling points. The values of the two criteria as well as the suggested sampling points are shown. An interactive version of this plot can be found at: https://www.tu-berlin.de/?204660.

Moreover, to proof the usage of the Q-criterion for the purpose of MBDoE, an optimization of the sampling points was conducted, using the experimental setting of the Michaelis-Menten kinetics example. The sampling points *t*_*bad*_ (0.75 and 1.5 h) were used as initial values. In every iteration, 500 Monte Carlo simulations were carried out, and the Q- as well as the A-criterion were used to guide the optimizer to optimal sampling points. As depicted in Figure 10, both criterions converge to their optimum after 20–30 iterations and suggest further measuring in similar time regions. This proofs that the Q-criterion is a valid criterion for the usage within MBDoE.

Since the A criterion may not be accessible due to the FIM being non-invertible in some cases with a high degree of non-linearity in the model, the Q criterion may be one of the few available options for performing MBDoE. In contrast to other methods this criterion is easy to use, as it does not require a deeper mathematical understanding nor does it use approximations like pseudoinverses to calculate the A-criterion (Shahmohammadi and McAuley, 2019). However, more research with complex models is necessary to further validate this usage.

## Conclusions

Despite the many advantages of planning experiments using MBDoE, the computation of linearized confidence intervals is still limiting its application in setups with complex systems and scarce data sets. We demonstrated that the confidence region approximated using the FIM is often only a poor description of the real parameter distribution. For this reason, we propose to perform Monte Carlo simulations to compute a more accurate distribution profile, despite the effort of additional computational burden.

In order to estimate the confidence of the estimated parameters and thus guide the MBDoE process, the FIM is commonly used. While this provides a rather good estimation in the vicinity of the optimal solution, this can be an issue especially in cases where the experimental information is scarce as shown in the biocatalytic reaction example. Here, Monte Carlo simulations provide an attractive alternative to calculate a more accurate distribution of the parameter confidence regions.

Finally, we propose a very simple and robust optimality criterion to obtain a convenient and scalar measure of the parameter distribution: The Q-criterion. The proposed criterion is based on Monte Carlo simulations making it computationally expensive. But in view of the rapid increase of computer power and considering that its parallelization is trivial (calculating 500 Monte Carlo simulations took 30 min on our workstation with 40 Intel Xeon E5-2640 2.4 GHz), it can be expected that this issue will cease to exist. Especially when performing MBDoE with highly non-linear models, where the FIM is not invertible, the Q-criterion can be used as a straightforward measure to optimize the experimental design.

## Author Contributions

NK, MG, TB, PN and MC contributed conception and design of the study. NK wrote the first draft of the manuscript. SK carried out the experiments. AS, SK, MG and MC wrote sections of the manuscript. All authors contributed to manuscript revision, read, and approved the submitted version.

### Conflict of Interest Statement

MG was employed by company DexLeChem GmbH. The remaining authors declare that the research was conducted in the absence of any commercial or financial relationships that could be construed as a potential conflict of interest.

## Figures and Tables

**Table:** 

*S*	Substrate concentration (g/L)
*P*	Product concentration (g/L)
*E*	Enzyme concentration (g/L)
*ES*	Enzyme substrate complex concentration (g/L)
*k*_1_, *k*_−1_, *k*_2_	Reaction rates [h^−1^]
ẋ(*t*)	Ordinary differential equations (ODE)
*x*(*t*)	Internal model states
*u*(*t*)	External stimuli
*r_*max*_*	Maximum specific reaction rate [h^−1^]
*K^*s*^*	Half saturation constant [g*L^−1^]
θ	Best currently available estimations of the set of parameters which must be determined (r_*max*_, K_*S*_)
ŷ(*t*)	Measured response variables
*y*(*t*)	Simulated response variables (substrate and product)
*g*(*x*(*t*))	Set of relations between the measured response variables ŷ(*t*) and the internal model states *x*(*t*)
η(*t*)	Measurement errors
*COV*	Parameter variance-covariance matrix
Σ_θ_	Covariance of the measuring noise
*n*_*mes*_	Number of measuring points
*n*_*resp*_	Number of responses
*n*_exp_	Number of experiments
*J*	Sum of squares of residuals
*y*^0^	Initial conditions for (*t* = *t*_0_ = 0)
*t*_*sp*_	Sampling times (*n* = 2)
$\bar{S}$	Sensitivity of the responses to parameter values
*FIM*	Fisher information matrix
φ	Experiment design vector, including sampling times, initial values and other experimental inputs for the future experiment
Ω	Measure of the FIM, i.e. optimal experimental design criterium
