# Supplementary material for: Monte Carlo Simulations for the Analysis of Non-linear Parameter Confidence Intervals in Optimal Experimental Design
Source: Front Bioeng Biotechnol. 2019 May 24;7:122. doi: 10.3389/fbioe.2019.00122 (PMC6543167; doi:10.3389/fbioe.2019.00122)
Supplement: Supplementary file 1 [file Table_1.docx]

Monte Carlo simulations for the analysis of nonlinear parameter confidence intervals in Optimal Experimental Design – Supplementary material

Niels Krausch^1^, Tilman Barz^3^, Annina Sawatzki^1^, Mathis Gruber^2^, Sarah Kamel^1^, Peter Neubauer^1^, Mariano Nicolas Cruz Bournazou^1*^

^1^ Department of Bioprocess Engineering, Department of Biotechnology, Technische Universität Berlin, Ackerstr. 76, ACK24, D-13355 Berlin, Germany

^2^ DexLeChem GmbH, Köpenicker Straße 325, House 11-12, 12555 Berlin, Germany

^3^ Department of Energy, Austrian Institute of Technology GmbH, Vienna, Austria

*** Correspondence:**Dr. Mariano Nicolas Cruz Bournazou
mariano.n.cruzbournazou@tu-berlin.de

Table 1 presents some of the most recent examples for model-based design of experiments in biotechnology, introducing standard and special criteria as well as showing their application in daily research.

Table 1: Recent examples for MbDoE in biotechnology

| **Reference:** | **Optimal design criterion:** | **Details:** |
| --- | --- | --- |
| (Faller et al., 2003) | D-optimal, E-optimal, modified E-optimal | Parameter identifiability and estimation applied to the MAP-kinase cascade |
| (Körkel et al., 2004) | Maximin (A-optimal) | Robust optimal experimental design for nonlinear dynamic systems (reaction of urethane); the maximin approach is solved using Taylor expansion to avoid semi-infinite optimization problems |
| (Flaherty et al.) | Maximin E-optimal | Robust experimental design using semidefinite programming, applied to Michaelis-Menten reaction model and calcium signal transduction model |
| (Yue et al., 2006) | Modified E-optimal | Identifiability analysis and measurement set selection via multivariate analysis applied to the IκB−NF−κB signaling pathway model |
| (Galvanin et al., 2007) | SV-optimality | Design of parallel experiments for a biomass fermentation of *Saccharomyces cerevisiae* |
| (Ataíde and Hitzmann, 2009) | A-optimal, E-optimal, D-optimal, equidistant experimental design | Comparison of different optimal experimental design criteria and traditional equidistant experimental design for parameter estimation of Michaelis-Menten kinetics |
| (He et al., 2010) | Maximin E-optimal, Bayesian | Comparison of maximin and bayesian experimental design (Michaelis-Menten enzyme kinetic pathway model, IκB−NF−κB signaling pathway model); study of the measurement set selection problem |
| (Yue et al., 2013) | D-optimal, E-optimal | Parameter estimation for enzyme kinetics |
| (Hoang et al., 2013) | A-optimal, D-optimal, E-optimal | Adaptive optimal sampling for parameter estimation using full discretization applied to a semi-batch reactor model |
| (Muñoz-Tamayo et al., 2014) | D-optimal | Parameter estimation for microalgae growth model |
| (Yu et al., 2015) | D-optimal, E-optimal | Parameter estimation by measurement set selection for an enzymatic biodiesel production |
| (Neddermeyer et al., 2016) | A-optimal | Model-validation and parameter estimation for the bacterium *Ralstonia eutropha* |
| (Cruz Bournazou et al., 2017) | A-optimal | Sliding window optimal experimental re-design for parameter estimation applied to parallel *Escherichia coli* fed-batch cultivations |
| (Galvanin et al., 2016) | A-optimal, j-optimal design | Joint model discrimination and parameter estimation for enzyme kinetics |
| (Chen et al., 2017) | Maximin D-optimal | Robust experimental design for parameter estimation using particle swarm optimization for enzyme kinetic inhibition models |
| (van Daele et al., 2017) | Maximin D-optimal | Iterative robust experimental design for parameter estimation for enzyme kinetics |

REFERENCES

Alper, J. S., and Gelb, R. I. (1990). Standard errors and confidence intervals in nonlinear regression: comparison of Monte Carlo and parametric statistics. *J. Phys. Chem.* 94, 4747–4751. doi: 10.1021/j100374a068

Ataíde, F., and Hitzmann, B. (2009). When is optimal experimental design advantageous for the analysis of Michaelis–Menten kinetics? *Chemometrics and Intelligent Laboratory Systems* 99, 9–18. doi: 10.1016/j.chemolab.2009.07.005

Audoly, S., Bellu, G., D'Angiò, L., Saccomani, M. P., and Cobelli, C. (2001). Global identifiability of nonlinear models of biological systems. *IEEE transactions on bio-medical engineering* 48, 55–65. doi: 10.1109/10.900248

Balsa-Canto, E., Banga, J. R., Alonso, A. A., and Vassiliadis, V. S. (2001). Dynamic optimization of chemical and biochemical processes using restricted second-order information. *Computers & Chemical Engineering* 25, 539–546. doi: 10.1016/S0098-1354(01)00633-0

Bard, Y. (1974). *Nonlinear parameter estimation*. New York: Acad. Press.

Barz, T., Sommer, A., Wilms, T., Neubauer, P., and Cruz Bournazou, M. N. (2018). Adaptive optimal operation of a parallel robotic liquid handling station. *IFAC-PapersOnLine* 51, 765–770. doi: 10.1016/j.ifacol.2018.04.006

Box, G. E. P., Hunter, W. G., and Hunter, J. S. (1978). *Statistics for experimenters: An introduction to design, data analysis, and model building*. New York: Wiley.

Buckland, S. T. (1984). Monte Carlo Confidence Intervals. *Biometrics* 40, 811. doi: 10.2307/2530926

Chen, P.-Y., Chen, R.-B., Tung, H.-C., and Wong, W. K. (2017). Standardized maximim D-optimal designs for enzyme kinetic inhibition models. *Chemometrics and intelligent laboratory systems : an international journal sponsored by the Chemometrics Society* 169, 79–86. doi: 10.1016/j.chemolab.2017.08.009

Cruz Bournazou, M. N., Barz, T., Nickel, D. B., Lopez Cárdenas, D. C., Glauche, F., Knepper, A., et al. (2017). Online optimal experimental re-design in robotic parallel fed-batch cultivation facilities. *Biotechnology and bioengineering* 114, 610–619. doi: 10.1002/bit.26192

Faller, D., Klingmüller, U., and Timmer, J. (2003). Simulation Methods for Optimal Experimental Design in Systems Biology. *SIMULATION* 79, 717–725. doi: 10.1177/0037549703040937

Flaherty, P., Arkin, A., and Jordan, M. I. “Robust design of biological experiments,” in *Advances in neural information processing*, 363–370.

Franceschini, G., and Macchietto, S. (2008). Model-based design of experiments for parameter precision: State of the art. *Chemical Engineering Science* 63, 4846–4872. doi: 10.1016/j.ces.2007.11.034

Galvanin, F., Cao, E., Al-Rifai, N., Gavriilidis, A., and Dua, V. (2016). A joint model-based experimental design approach for the identification of kinetic models in continuous flow laboratory reactors. *Computers & Chemical Engineering* 95, 202–215. doi: 10.1016/j.compchemeng.2016.05.009

Galvanin, F., Macchietto, S., and Bezzo, F. (2007). Model-Based Design of Parallel Experiments. *Industrial & Engineering Chemistry Research* 46, 871–882. doi: 10.1021/ie0611406

Glauche, F., Pilarek, M., Bournazou, M. N. C., Grunzel, P., and Neubauer, P. (2017). Design of experiments-based high-throughput strategy for development and optimization of efficient cell disruption protocols. *Eng. Life Sci.* 17, 1166–1172. doi: 10.1002/elsc.201600030

Goujot, D., Meyer, X., and Courtois, F. (2012). Identification of a rice drying model with an improved sequential optimal design of experiments. *Journal of Process Control* 22, 95–107. doi: 10.1016/j.jprocont.2011.10.003

Guisasola, A., Baeza, J. A., Carrera, J., Sin, G., Vanrolleghem, P. A., and Lafuente, J. (2006). The Influence of Experimental Data Quality and Quantity on Parameter Estimation Accuracy. *Education for Chemical Engineers* 1, 139–145. doi: 10.1205/ece06016

He, F., Brown, M., and Yue, H. (2010). Maximin and Bayesian robust experimental design for measurement set selection in modelling biochemical regulatory systems. *International Journal of Robust and Nonlinear Control* 20, 1059–1078. doi: 10.1002/rnc.1558

Heineken, F. G., Tsuchiya, H. M., and Aris, R. (1967). On the mathematical status of the pseudo-steady state hypothesis of biochemical kinetics. *Mathematical Biosciences* 1, 95–113. doi: 10.1016/0025-5564(67)90029-6

Hemmerich, J., Noack, S., Wiechert, W., and Oldiges, M. (2018). Microbioreactor Systems for Accelerated Bioprocess Development. *Biotechnology journal* 13, e1700141. doi: 10.1002/biot.201700141

Hoang, M. D., Barz, T., Merchan, V. A., Biegler, L. T., and Arellano-Garcia, H. (2013). Simultaneous solution approach to model-based experimental design. *AIChE J.* 59, 4169–4183. doi: 10.1002/aic.14145

Holmberg, A. (1982). On the practical identifiability of microbial growth models incorporating Michaelis-Menten type nonlinearities. *Mathematical Biosciences* 62, 23–43. doi: 10.1016/0025-5564(82)90061-X

Kamel, S., Weiß, M., Klare, H. F.T., Mikhailopulo, I. A., Neubauer, P., and Wagner, A. (2018). Chemo-enzymatic synthesis of α-d-pentofuranose-1-phosphates using thermostable pyrimidine nucleoside phosphorylases. *Molecular Catalysis* 458, 52–59. doi: 10.1016/j.mcat.2018.07.028

Kennard, R. W., and Stone, L. A. (1969). Computer Aided Design of Experiments. *Technometrics* 11, 137–148. doi: 10.1080/00401706.1969.10490666

Körkel, S., Kostina, E., Bock, H. G., and Schlöder, J. P. (2004). Numerical methods for optimal control problems in design of robust optimal experiments for nonlinear dynamic processes. *Optimization Methods and Software* 19, 327–338. doi: 10.1080/10556780410001683078

Kostina, E., and Nattermann, M. (2015). Second-Order Sensitivity Analysis Of Parameter Estimation Problems. *Int. J. UncertaintyQuantification* 5, 209–231. doi: 10.1615/Int.J.UncertaintyQuantification.2015010312

Kravaris, C., Hahn, J., and Chu, Y. (2013). Advances and selected recent developments in state and parameter estimation. *Computers & Chemical Engineering* 51, 111–123. doi: 10.1016/j.compchemeng.2012.06.001

Kreutz, C., Raue, A., and Timmer, J. (2012). Likelihood based observability analysis and confidence intervals for predictions of dynamic models. *BMC systems biology* 6, 120. doi: 10.1186/1752-0509-6-120

López C, D. C., Barz, T., Peñuela, M., Villegas, A., Ochoa, S., and Wozny, G. (2013). Model-based identifiable parameter determination applied to a simultaneous saccharification and fermentation process model for bio-ethanol production. *Biotechnology progress* 29, 1064–1082. doi: 10.1002/btpr.1753

López C., D. C., Barz, T., Körkel, S., and Wozny, G. (2015). Nonlinear ill-posed problem analysis in model-based parameter estimation and experimental design. *Computers & Chemical Engineering* 77, 24–42. doi: 10.1016/j.compchemeng.2015.03.002

Mikhailopulo, I. A., and Miroshnikov, A. I. (2013). Some recent findings in the biotechnology of biologically important nucleosides. *Biotechnologia Acta* 6, 328–353.

Miroshnikov, A. I., Esipov, R. S., Muravyova, T.'y. I., Konstantinova, I. D., Fateev, I. V., and Mikhailopulo, I. A. (2010). A New Strategy for the Synthesis of Nucleosides: One-Pot Enzymatic Transformation of D-Pentoses into Nucleosides. *TOPROCJ* 1, 98–102. doi: 10.2174/22102892010010100098

Moles, C. G., Mendes, P., and Banga, J. R. (2003). Parameter estimation in biochemical pathways: a comparison of global optimization methods. *Genome research* 13, 2467–2474. doi: 10.1101/gr.1262503

Muñoz-Tamayo, R., Martinon, P., Bougaran, G., Mairet, F., and Bernard, O. (2014). Getting the most out of it: Optimal experiments for parameter estimation of microalgae growth models. *Journal of Process Control* 24, 991–1001. doi: 10.1016/j.jprocont.2014.04.021

Muñoz-Tamayo, R., Puillet, L., Daniel, J. B., Sauvant, D., Martin, O., Taghipoor, M., et al. (2018). Review: To be or not to be an identifiable model. Is this a relevant question in animal science modelling? *Animal : an international journal of animal bioscience* 12, 701–712. doi: 10.1017/S1751731117002774

Neddermeyer, F., Marhold, V., Menzel, C., Krämer, D., and King, R. (2016). Modelling the production of soluble hydrogenase in Ralstonia eutropha by on-line optimal experimental design**This work was supported by the DFG in the framework of the cluster of excellence UniCat. *IFAC-PapersOnLine* 49, 627–632. doi: 10.1016/j.ifacol.2016.07.238

Neubauer, P., Cruz, N., Glauche, F., Junne, S., Knepper, A., and Raven, M. (2013). Consistent development of bioprocesses from microliter cultures to the industrial scale. *Eng. Life Sci.* 13, 224–238. doi: 10.1002/elsc.201200021

Nickel, D. B., Cruz-Bournazou, M. N., Wilms, T., Neubauer, P., and Knepper, A. (2017). Online bioprocess data generation, analysis, and optimization for parallel fed-batch fermentations in milliliter scale. *Eng. Life Sci.* 17, 1195–1201. doi: 10.1002/elsc.201600035

Oliver Lindner, P. F., and Hitzmann, B. (2006). Experimental design for optimal parameter estimation of an enzyme kinetic process based on the analysis of the Fisher information matrix. *Journal of theoretical biology* 238, 111–123. doi: 10.1016/j.jtbi.2005.05.016

Pronzato, L., and Pázman, A. (2013). *Design of Experiments in Nonlinear Models*. New York, NY: Springer New York.

Pugmire, M. J., and Ealick, S. E. (2002). Structural analyses reveal two distinct families of nucleoside phosphorylases. *Biochem. J.* 361, 1–25. doi: 10.1042/bj3610001

Raue, A., Kreutz, C., Maiwald, T., Bachmann, J., Schilling, M., Klingmüller, U., et al. (2009). Structural and practical identifiability analysis of partially observed dynamical models by exploiting the profile likelihood. *Bioinformatics (Oxford, England)* 25, 1923–1929. doi: 10.1093/bioinformatics/btp358

Sacks, J., Welch, W. J., Mitchell, T. J., and Wynn, H. P. (1989). Design and analysis of computer experiments. *Statistical science*, 409–423.

Sawatzki, A., Hans, S., Narayanan, H., Haby, B., Krausch, N., Sokolov, M., et al. (2018). Accelerated Bioprocess Development of Endopolygalacturonase-Production with Saccharomyces cerevisiae Using Multivariate Prediction in a 48 Mini-Bioreactor Automated Platform. *Bioengineering* 5. doi: 10.3390/bioengineering5040101

Schenkendorf, R., Kremling, A., and Mangold, M. (2009). Optimal experimental design with the sigma point method. *IET systems biology* 3, 10–23. doi: 10.1049/iet-syb:20080094

Schenkendorf, R., Xie, X., Rehbein, M., Scholl, S., and Krewer, U. (2018). The Impact of Global Sensitivities and Design Measures in Model-Based Optimal Experimental Design. *Processes* 6, 27. doi: 10.3390/pr6040027

Silvey, S. D. (1980). *Optimal Design: An Introduction to the Theory for Parameter Estimation*. Dordrecht: Springer.

Sin, G., Gernaey, K. V., Neumann, M. B., van Loosdrecht, M. C. M., and Gujer, W. (2009). Uncertainty analysis in WWTP model applications: a critical discussion using an example from design. *Water Research* 43, 2894–2906. doi: 10.1016/j.watres.2009.03.048

Skanda, D., and Lebiedz, D. (2010). An optimal experimental design approach to model discrimination in dynamic biochemical systems. *Bioinformatics (Oxford, England)* 26, 939–945. doi: 10.1093/bioinformatics/btq074

Stephanopoulos, G., and Reklaitis, G. V. (2011). Process systems engineering: From Solvay to modern bio- and nanotechnology. *Chemical Engineering Science* 66, 4272–4306. doi: 10.1016/j.ces.2011.05.049

Szeker, K., Zhou, X., Schwab, T., Casanueva, A., Cowan, D., Mikhailopulo, I. A., et al. (2012). Comparative investigations on thermostable pyrimidine nucleoside phosphorylases from Geobacillus thermoglucosidasius and Thermus thermophilus. *Journal of Molecular Catalysis B: Enzymatic* 84, 27–34. doi: 10.1016/j.molcatb.2012.02.006

Tarantola, A. (2005). *Inverse problem theory and methods for model parameter estimation*. Philadelphia, Pa. SIAM - Soc. for Industrial and Applied Math.

Telen, D., Nimmegeers, P., and van Impe, J. (2018). Uncertainty in optimal experiment design: comparing an online versus offline approaches. *IFAC-PapersOnLine* 51, 771–776. doi: 10.1016/j.ifacol.2018.04.007

Tozzi, M. G., Camici, M., Mascia, L., Sgarrella, F., and Ipata, P. L. (2006). Pentose phosphates in nucleoside interconversion and catabolism. *The FEBS journal* 273, 1089–1101. doi: 10.1111/j.1742-4658.2006.05155.x

van Daele, T., Gernaey, K. V., Ringborg, R. H., Börner, T., Heintz, S., van Hauwermeiren, D., et al. (2017). Application of iterative robust model-based optimal experimental design for the calibration of biocatalytic models. *Biotechnology progress* 33, 1278–1293. doi: 10.1002/btpr.2515

Vanrolleghem, P. (1995). Practical identifiability of a biokinetic model of activated sludge respiration. *Water Research* 29, 2561–2570. doi: 10.1016/0043-1354(95)00105-T

Vassiliadis, V. S., Canto, E. B., and Banga, J. R. (1999). Second-order sensitivities of general dynamic systems with application to optimal control problems. *Chemical Engineering Science* 54, 3851–3860. doi: 10.1016/S0009-2509(98)00432-1

Velten, K. (2009). *Mathematical modeling and simulation: Introduction for scientists and engineers*. Weinheim Germany: Wiley-VCH.

Walter, E., and Pronzato, L. (1990). Qualitative and quantitative experiment design for phenomenological models—A survey. *Automatica* 26, 195–213. doi: 10.1016/0005-1098(90)90116-Y

Yehia, H., Kamel, S., Paulick, K., Wagner, A., and Neubauer, P. (2017). Substrate spectra of nucleoside phosphorylases and their potential in the production of pharmaceutically active compounds. *Current pharmaceutical design*. doi: 10.2174/1381612823666171024155811

Yu, H., Yue, H., and Halling, P. (2015). Optimal Experimental Design for an Enzymatic Biodiesel Production System. *IFAC-PapersOnLine* 48, 1258–1263. doi: 10.1016/j.ifacol.2015.09.141

Yue, H., Brown, M., Knowles, J., Wang, H., Broomhead, D. S., and Kell, D. B. (2006). Insights into the behaviour of systems biology models from dynamic sensitivity and identifiability analysis: a case study of an NF-kappaB signalling pathway. *Molecular bioSystems* 2, 640–649. doi: 10.1039/B609442B

Yue, H., Halling, P., and Yu, H. (2013). Model Development and Optimal Experimental Design of A Kinetically Controlled Synthesis System. *IFAC Proceedings Volumes* 46, 327–332. doi: 10.3182/20131216-3-IN-2044.00034
